# Graphene-augmented nanofiber scaffolds demonstrate new features in cells behaviour

**DOI:** 10.1038/srep30150

**Published:** 2016-07-22

**Authors:** Jekaterina Kazantseva, Roman Ivanov, Michael Gasik, Toomas Neuman, Irina Hussainova

**Affiliations:** 1 Cellin Technologies LLC, Tallinn, Estonia; 2 Department of materials engineering, Tallinn University of Technology, Tallinn, Estonia; 3 School of Chemistry Technology, Aalto University Foundation, Espoo, Finland; 4 Protobios LLC, Tallinn, Estonia; 5 ITMO University, St. Petersburg, Russian Federation

## Abstract

Three-dimensional (3D) customized scaffolds capable to mimic a native extracellular matrix open new frontiers in cells manipulation and advanced therapy. The major challenge is in a proper substrate for *in vitro* models on engineered scaffolds, capable to modulate cells differentiation. Here for the first time we demonstrate novel design and functionality of the 3D porous scaffolds of aligned, self-assembled ceramic nanofibers of ultra-high anisotropy ratio (~10^7^), augmented into graphene shells. This unique hybrid nano-network allows an exceptional combination of selective guidance stimuli of stem cells differentiation, immune reactions variations, and local immobilization of cancer cells, which was not available before. The scaffolds were shown to be able to direct human mesenchymal stem cells (important for stimulation of neuronal and muscle cells) preferential orientation, to suppress major inflammatory factors, and to localize cancer cells; all without additions of specific culture media. The selective downregulation of specific cytokines is anticipated as a new tool for understanding of human immune system and ways of treatment of associated diseases. The effects observed are self-regulated by cells only, without side effects, usually arising from use of external factors. New scaffolds may open new horizons for stem cells fate control such as towards axons and neurites regeneration (Alzheimer’s disease) as well as cancer therapy development.

Regenerative medicine and tissue engineering as well as cell therapy represent a breakthrough change in paradigm in healthcare compared to traditional pharmacology and implantology^1^. The role of proper *in vitro* protocols and systems for cells and drugs research is vital in creation of cost-effective and scientifically validated treatment methods, at the same time implementing expensive and scattered animal studies (known as “refinement, reduction, replacement” – the “3R”), and long, even more expensive clinical trials. For realization of efficient cells adhesion, proliferation, morphogenesis and differentiation, scaffolds should properly mimic natural *in vivo* microenvironments and signals, and offer correct local conditions needed for the regulation of vital cellular functions^1^,^2^,^3^,^4^. Besides biochemical factors, the surface and topography of a scaffold affects greatly stem cell fate^3^ (for example, a fibrous scaffold was found to increase neural stem cell oligodendrocyte differentiation as well as greatly improve neurite extension and gene expression for neural markers^4^). As another example, scaffolds with conductive surfaces can promote human mesenchymal stem cells (hMSC) differentiation towards electro-active lineages^3^,^4^,^5^, opening new scenarios for regeneration of neural, cardiac and similar tissues, capable to assist drug research *in vitro*.

Recently graphene became an important material in cellular studies and tissue engineering applications due to its extraordinary properties^6^,^7^,^8^,^9^,^10^,^11^. However, non-modified graphene materials have shown increased cellular toxicity and a high degree of agglomeration in aqueous medium^12^, which limits their direct practical applications in scaffolds. To overcome these limitations, graphene is usually subjected to a substantial chemical modification (introduction of oxy- and hydroxyl groups and probably grafting of some organic molecules) to be combined with desired properties and bio-functionality^13^.

For the first time, we report here graphene-augmented inorganic nanofibres (GAIN) scaffolds with tailored mechanical anisotropy, fibres size, orientation and porosity. These 3D hybrid GAIN scaffolds are shown to be biocompatible and robust, well suitable for evaluation of cell studies, allowing previously unavailable features in the cell culture environments due to controlled ultra-high 3D anisotropy and surface variations.

## Scaffolds for cells cultures

**Figure 1:**
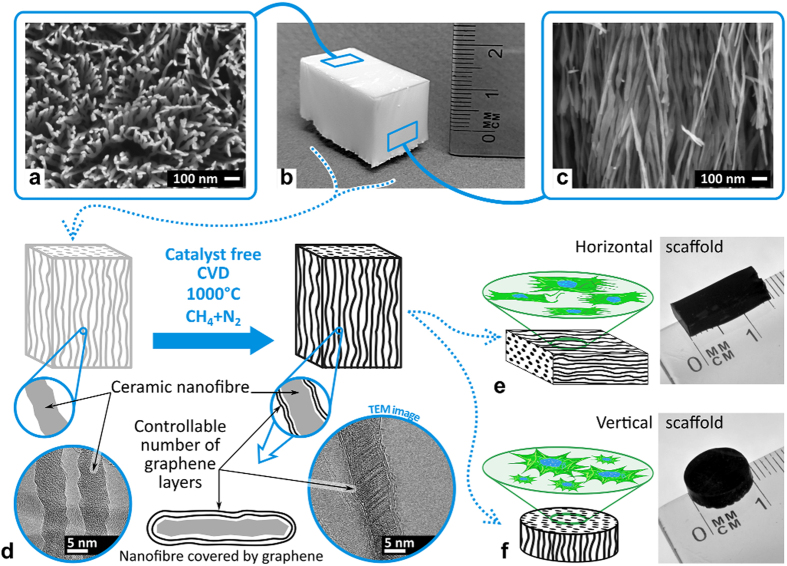
(**a**–**c**) The images of the graphene-augmented inorganic nanofibres (GAIN) network: (**a**) the top view (SEM) of GAIN revealing high porosity; (**b**) the network of the highly oriented nanofibres held together by weak hydrogen bonding; (**c**) the side view (SEM) of GAIN showing alignment of the fibres. (**d**) The schematic of the process of a single-step catalyst-free CVD of controllable number of *“in-situ*” self-assembly of graphene layers onto the surface of fibres using CH_4_ gas as a source of carbon. (**e**) The GAIN scaffold with horizontally oriented fibres allowing directional orientation (estimated tangential compressive elastic modulus 200–400 GPa along the fibres and ~1–5 kPa across the nanofibres network) and altering morphology of different types of cells. (**f**) The GAIN scaffold with vertically oriented fibres allowing development of mixed tumour *in vitro* models 2D/3D configurations for inductive and conductive features, accomplish the selective propagation of tumour cells.

The unique self-assembled metal oxide fibres network with an average single-fiber diameter of ~40 nm (TEM, HRSEM) and length of 2–10 cm with 85–95% oriented porosity (BET; mercury porosimetry) was produced using bottom-up approach of controlled liquid phase oxidation^14^ and employed as the substrate for graphene shells growth in a single-step process of novel catalyst-free chemical vapor deposition (CVD) at 1000 °C and atmospheric pressure using a mixture of nitrogen (N_2_) and methane (CH_4_) gases, Fig. 1. Recently, for the first time we demonstrated that alumina nanofibers can successfully serve as a substrate for the direct growth of few-layered graphene close shells around the dielectric fibre^15^. On the contrary to many other studies, graphene on the nanofibres was not functionalized in any way and became augmented to the underlying oxide fiber surface during the manufacturing process.

For cells behavior analysis (stem cells proliferation and fate; cancer cells attachment and growth; gene expressions variations, etc.), many properties of 3D scaffolds are essential. For example, substrate stiffness and topology are well known to modulate primary cells shape and morphology with conditions required for further specific differentiation. However, their explicit interactions of material single features with cells and ECM system are usually too complex to allow separation of specific parameters contribution to a reliable extent.

## Stem cells evolution

**Figure 2:**
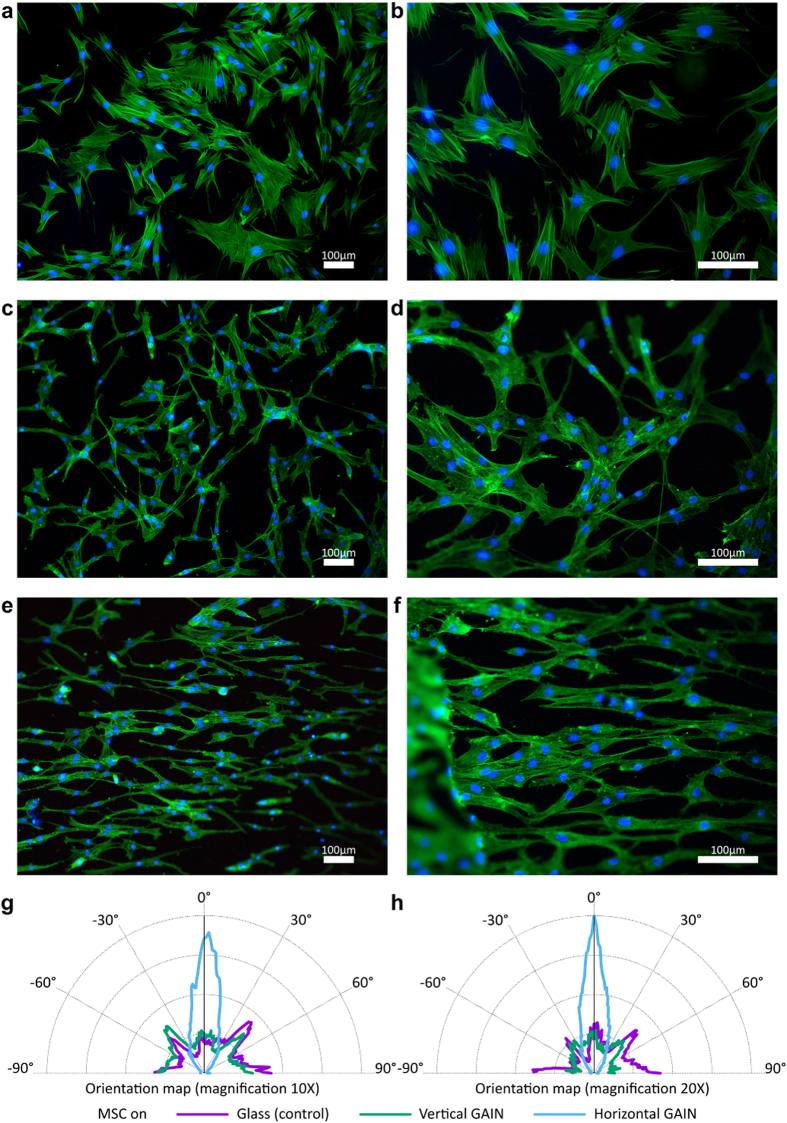
Fluorescence microscopy images (two different magnifications) of hMSC on control (glass, **a**,**b**), GAIN vertical (**c**,**d**) and GAIN horizontal (**e**,**f**) scaffolds. The preferential orientation comparison (**g**,**h**) indicates several times higher anisotropy for GAIN-seeded hMSC.

Firstly, we explored potential use of GAIN scaffolds for tissue engineering applications by studying *in vitro* the growth, proliferation and especially orientation of adipose-derived hMSC. The morphology, adhesion and distribution of viable hMSC after 3 days of culture on horizontally oriented GAIN scaffolds and on control are demonstrated in Fig. 2. GAIN scaffolds were seen clearly guiding cells to line up along the fibres with a spindle-like shape and more than usually elongated cytoplasmic lamellipodia extensions. High aspect ratio cellular orientations are developed throughout the GAIN scaffold (Fig. 2b) allowing directional connection between individual cells and formation of cells network. Thus non-modified, augmented graphene on GAIN scaffolds does not impede the normal growth of stem cells. Furthermore, elongated morphology of hMSC and its high polarization (Fig. 2c) create pre-requisites for preferential specific (e. g. neuronal or myogenic) lineage differentiation.

Elastic properties of the scaffold substrate (stiffness and viscoelasticity) play a definitive role in stem cells behaviour^16^. For example, elastic modulus range of ~1 kPa is known to promote differentiation especially into neural cells and their growth^17^, as it is close to the neural and brain tissue stiffness. For GAIN scaffolds, the tangential elastic modulus anisotropy (Fig. 1) is about 400 GPa/5 kPa = 80·10^6^, which variation range covers elasticity of actin filaments involved in cells adhesion and taxis (the elastic modulus of F-actin was reported^14^ to be close to 2 GPa). At the same time, diameter of the nanofibres in GAIN scaffolds (Fig. 1) is ~40 nm (500–1000 times smaller than average cell size), but compatible to the size^14^ of actin filaments spacing (20–30 nm). Thus, one cell filament can be bound to exactly one nanofiber in orthogonal direction, but many filaments can attach to one nanofiber along its longer length. In terms of adhesion process, cell membrane compliance and elasticity would cause substantially larger displacement of nanofibres in perpendicular direction whereas in parallel to nanofibres the cell membrane would face substantially larger stress and therefore undergo much higher deformation.

Whereas response of stem cells to different substrate stiffness was studied earlier, to our best knowledge, the effect of such ultra-high local modulus anisotropy in 3D nanofibers was not previously reported. One of the reasons is that most of nanofibre-based scaffolds are randomly oriented or loosely packed, making a 3D nanostructure with not such stiffness anisotropy. Many aligned fibrous scaffolds are made of polymer fibres, which intrinsic elastic modulus is less than oxide ceramics by few orders of magnitude. The effect observed at GAIN scaffolds differs also substantially from known nanotopology studies, where nano-grooves or other patterns are known to support cell alignment, but without huge local differences in substrate stiffness anisotropy.

We suggest that “durotaxis” effect^16^,^17^,^18^ drives hMSC along the stiffer scaffold direction, whereas orthogonally it leads to local confining of parallel nanofibres due to traction force expressed by the cell membrane. Dynamically crawling cell may create periodic contraction and expansion of nanofibres across the cell main direction. Such “nanopump” in GAIN scaffolds allows more intensive local media flow ensuring better nutrients and oxygen supply thus supporting the cell own metabolic processes.

## Tumour models

**Figure 3:**
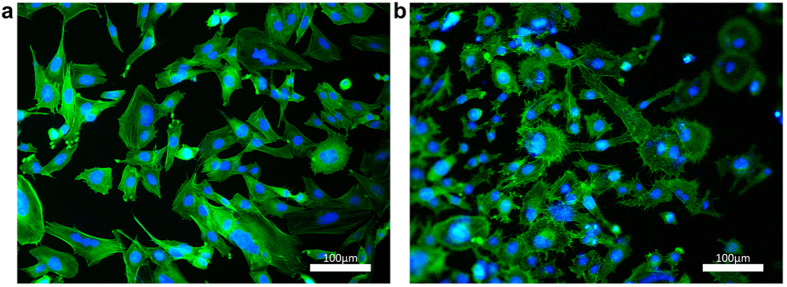
Fluorescent images of breast cancer cell line MDA-MB 231 showing the morphological changes of cells grown on control glass (**a**) and horizontal GAIN scaffolds (**b**), with evident increased number of microspikes and initial stages of cytoplasm infiltration into the scaffolds supposing high membrane activity.

The second application of GAIN scaffolds was demonstrated for cancer cells growth. Breast cancer cell line MDA-MB231 was seeded on the vertical and horizontal fibres of the GAIN scaffolds (Fig. 1). Cancer cells cultured on GAIN possess extended microspikes and actin-rich filopodia protrusions suggesting high level of membrane activity and malignant migratory cancer phenotype (Fig. 3). The initial signs of cancer cell infiltration (and hence local immobilization of the cells) were detected on vertical fibres allowing suggestion of 3D cancer model. Observed cancer cell infiltration, their clearly reduced mobility and extended filopodia formation, GAIN scaffold creates more representative phenotype to obtain 3D *in vitro* cancer model, mimicking tumour genesis more accurately.

The unique composition and structure of GAIN scaffolds creates a microenvironment, which fundamentally contrasts with such for tumour cells grown on 2D morphology. These effects assertively cause different expression of oncogenes and tumour suppressor genes^19^. In general, the behaviour of cancer cells on GAIN scaffolds differs from 2D culture and can be used as a tool for anti-cancer drug development or in cancer stem cell (CSC) studies, as the proportion of CSC has been shown significantly greater for 3D system^20^.

## Inflammatory response

In the third challenge, the inflammatory response of GAIN scaffolds was considered. Immunological profiling of any material under the influence of *in vivo* or *in vitro* environment is an important content of biocompatibility evaluation and has a critical value for future clinical translation. In addition to hMSC, this was also assessed with peripheral blood mononuclear cells (PBMCs) as they are an important part of the human peripheral immune system, responsible for transforming of the multitude of external stimuli to generate an adaptive immune response.

**Figure 4:**
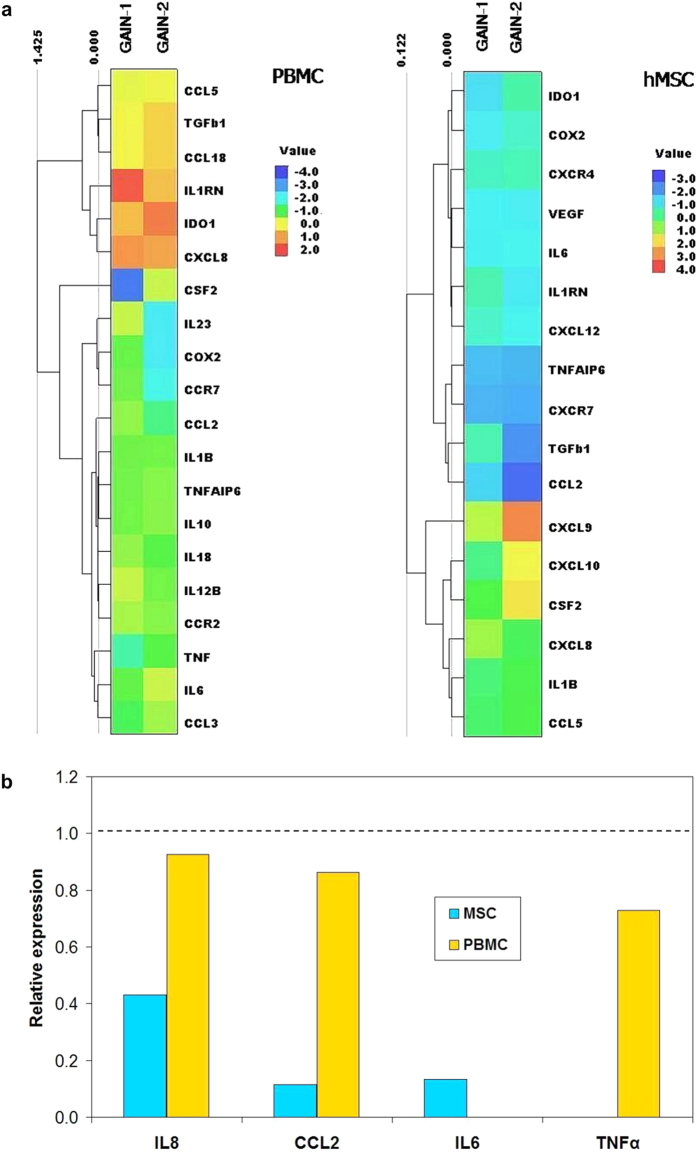
The immunologic profile impact on GAIN scaffolds for hMSCs and PBMCs (incubated for 24 h) with the inflammatory signature compared to the cells without the scaffold (control). (**a**) mRNA expression of genes responsible for immune reaction (quantitative real-time polymerase chain reaction, RT-qPCR). The results are represented as a heat map for two independent set of measurements on separate GAIN scaffolds, revealing simultaneously row hierarchical cluster structure in a data matrix with the location of the respective tiles near each other telling about similarity in the respective gene expression groups. (**b**) Secretion of some important cytokines (enzyme-linked immunosorbent assay, ELISA), normalized to the control cells expression and displayed as a histogram, where dashed line corresponds to the expression level of cytokines in the control cells.

Observed unimpaired nuclei morphology of hMSC (Fig. 2) indicates no cytotoxicity for GAIN scaffolds. Then, hMSC and PBMC were grown on the GAIN scaffolds and tissue culture plastic (control) *in vitro,* and their inflammatory signatures were compared (Fig. 4). We analyzed the mRNA expression of genes involved in the immune reaction and a secretion of cytokines (IL1ß, TNFα, IFNγ, IL6, IL8, IL2, IL4, IL12ß, and CCL2).

For PBMC, clear downregulation of pro-inflammatory *TNF, IL1B, IL12B, IL6*, *CCL2* and *COX2* cytokines was detected, demonstrating the strong GAIN ability to modulate immune response. Among the upregulated inflammatory genes in GAIN microenvironment *CCL18, IL1RN, IDO1* and *TGFß1* factors were observed. These factors commonly participate in anti-inflammatory response, pointing out to the possible immunomodulating effect of the GAIN scaffolds. For hMSCs, the promoted mRNA expression of *CXCL8, CXCL9, CXCL10* and *CSF2*, the chemokines participating in neutrophil, monocyte or leukocyte trafficking, were observed, indicating the possible changes in chemotaxis in response to the GAIN. Additionally, reduced expression of *COX2, CCL2* and *IL6* cytokines indicates a good immune tolerance of the GAIN material.

Analysis of cytokines secretion by ELISA (Fig. 4b) also demonstrated the reduced levels of TNFα, IL6, IL8/CXCL8, and CCL2 expression and total absence of secretion of pro-inflammatory IL1ß, IFNγ, IL2, IL4 and IL12 cytokines by both cell types assuming immuno-indifferent impact of the materials.

The main transcript induced in response to scaffold microenvironment for both cell types was *CXCL8*, but the secretion of this cytokine in medium was reduced comparing the control. Although *CXCL8* can be involved into the initiation of acute inflammatory processes, the global immunologic signature was undoubtedly immuno-tolerant. The heightened expression of IL1RN and IDO1 together with the elevated levels of CCL18 and TGFß1 in PBMCs (Fig. 4a) also has confirmed the immunoregulatory function of the GAIN.

## Conclusions

Unique properties of GAIN scaffolds provide an improved capacity in enhancing hMSC alignment without additional manipulations. GAIN provides a suitable engineering microenvironment of nanotopological features, transducting physical cues with a high positive impact on a possible cell differentiation fate, which is of a paramount importance for cell therapy. Moreover, GAIN scaffolds positively affect immunological behaviour of hMSC and PBMC, with specific regulation of cytokines, revealing immuno-tolerant nature of the materials. The unique behaviour of studied breast cancer cells on GAIN scaffolds and their immobilization *in loco* open new frontiers in advanced materials for 3D tumour growth modelling systems and anticancer therapy development.

## Methods

### Cell cultures

Human MSCs were obtained from freshly isolated subcutaneous adipose tissue and characterized as previously reported^21^. Human breast cancer cell line MDA-MB 231 was purchased from ATCC. Cells were grown in DMEM with 10% FBS, 1 mg/ml penicillin and 0.1 mg/ml streptomycin at 37 °C in 5% CO_2_. GAIN scaffolds were pre-treated for three days before the cells seeding by complete medium with changing the medium for fresh every 24 hours to saturate them by active components adsorbed from liquid phase.

### Cells staining and visualisation

For visualisation of adipose-derived hMSCs and MDA-MB 231 on GAIN scaffold, we used specific to filamentous actin (F-actin) phalloidin tagged by FITC (Sigma). For hMSCs, pooled cells from three individuals with passage number below 5 were seeded on the scaffolds in 12-well plate (4 × 10^4^ cells per well). For MDA-MB 231, 5 × 10^4^ cells were added to the each well with scaffolds. Similar cells grown on a flat glass at the same density and cell culture conditions were considered as the controls. The cells were fixed by 4% PFA at 48 h after seeding, washed by PBS and permeabilized by 0.3% TRITON X-100 in PBS for 5 minutes. Phalloidin-FITC (1:100) staining lasted for 18 h at 4 °C for GAIN scaffolds and 2 h at RT for controls. To stain the cells nucleus, cells were incubated for 10 minutes with Hoechst 33342 (Invitrogen, 1 μg/ml). After a final wash, the phalloidin-stained cells were analysed by Nikon Eclipse 80i microscope.

### Cells orientation analysis

**Figure 5:**
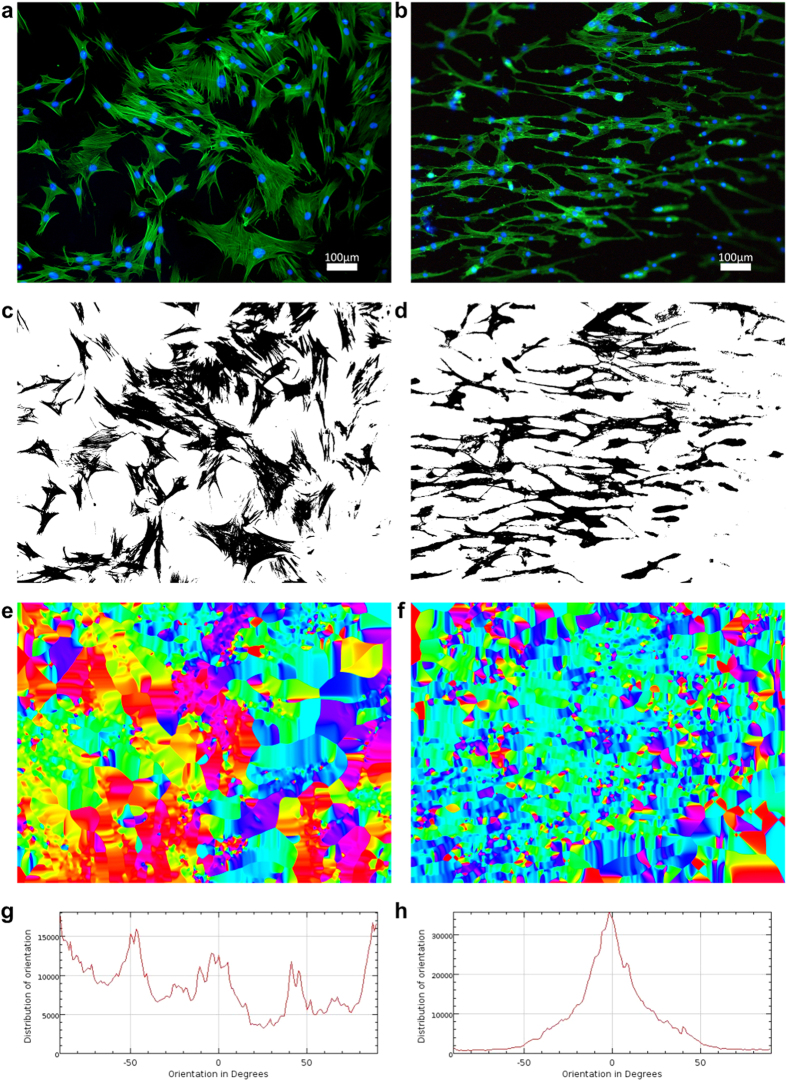
Original fluorescent microscopy pictures (**a**,**b**), processed binary images (**c**,**d**), orientation texture maps (**e**,**f**) with colour differences (rainbow spectrum from red to violet) indicate preferential orientation and these orientation distribution summaries (sum of distances in μm) plots (**g**,**h**) from −90 to 90° (clockwise). Images made on control (**a**,**c**,**e**,**g**) and horizontal GAIN (**b**,**d**,**f**,**h**) scaffolds.

Since hMSC are capable of self-renewal and differentiation *in vitro* into multiple cell lineages depending on growth factors, microenvironment, and availability of substrates with different topography and rigidity^22^, the orientation of the cells is an important property to achieve efficient differentiation while eliminating the potential variable side effects from external growth factors and inducers in the culture media. Calculation of the orientations of hMSC seeded on control and GAIN scaffolds was used with “ImageJ” software (version 1.50g, National Institute of Health, USA) and Orientation-J Distribution plug-in. Original microscope images (Fig. 5a) were converted into 8-bit colour images and the pixels/distance ratio was calibrated based on the microscope camera bar. The images were threshold first by HSV colour and then by brightness into binary images using Li algorithm (Fig. 5b). The orientation parameters were calculated with 5 pixel Gaussian window size and approximation with a cubic spline gradient. The resulting orientation texture maps are shown in Fig. 5c, and respective rotation angle span plots in Fig. 5d. Finally, these data were treated with SigmaPlot software (Systat GmbH, Germany) into polar form as shown in Fig. 2.

### Immunological analysis

PBMCs from healthy donors were isolated using Ficoll-Paque gradient fractionation. In total 2·10^6^ cells were used for each analysis. RNAs were extracted directly from scaffolds by TRIzol^®^ (Ambion) reagent following 24 h cells growth on GAIN scaffold, according to the manufacturer’s recommendations. cDNAs were synthesised from DNase-treated (Ambion) RNA by RevertAid Reverse Transcriptase (Thermo Fisher Scientific) with addition of RiboLock (Thermo Fisher Scientific) according to the manufacturer’s recommendations. cDNA quality was verified by RT-PCR by using GAPDH primers and HOT FIREpol^®^ Master Mix (Solis Biodyne, Estonia). RT-qPCR was performed in triplicates using EvaGreen qPCR mix plus no Rox (Solis Biodyne, Estonia) and the LightCycler^®^ 480 Real-Time PCR System (Roche Applied Science). The fold of change was calculated relatively to the control (cells grown without scaffolds) after normalisation to GAPDH expression, using 2-ΔΔCt method (double difference of Ct). The values are respectively ΔCt = Ct(gene of interest) − Ct(GAPDH), and ΔΔCt = ΔCt(treated) − ΔCt(control). For visualization, the data were normalized to the cells grown without scaffold materials (control), converted to log scale and represented as a heat map (Fig. 4a; LionSolver 2.1, Reactive Search s.r.l., Italy). The represented data show values of two independent analyses normalized to the levels of cytokine expression for cells grown without GAIN scaffolds. Each tile is shaded on a colour scale to represent the value of the corresponding element of the data matrix. The rows of the data matrix are ordered such that similar rows are near each other, through hierarchical clustering using Euclidean distance (the distance between the two vectors in the feature space). The minimal mapping error is achieved by minimizing sum of coordinates normalized with respect to the maximum and minimum along each dimension (in this case, decimal logarithm of relative expressions).

### Secretome ELISA analysis

Cell culture media was collected and soluble factors expression was analysed 24 h after initiation of cell culture. The levels of IL6, IL8/CXCL8, CCL2, IL1B, IL2, IL4, IL12, TNFα and IFNγ secreted into the growth medium were measured using Human IL-6 DuoSet ELISA Development Kit (R&D System, Wiesbaden, Germany), Human IL-8 Standard ABTS ELISA Development Kit (Peprotech, Rock Hill, NJ, USA), Human CCL2/MCP-1 DuoSet (R&D System), Human IL-1β/IL-1F2 DuoSet ELISA Development Kit (R&D System, Wiesbaden, Germany), Human IL-2 ELISA Development Kit (Peprotech, Rock Hill, NJ, USA), Human IL-4 Standard ABTS (PeproTech, Rock Hill, NJ, USA), Human IL-12 ELISA Development Kit (Peprotech, Rock Hill, NJ, USA), Human TNFα ELISA Development Kit (Peprotech, Rock Hill, NJ, USA), Human IFN-γ ELISA Development Kit (Peprotech, Rock Hill, NJ, USA), and Human Standard ABTS ELISA Development Kit (Peprotech), respectively. The ELISA analysis was performed using high binding ELISA plates (Greiner BioOne) at RT according to the manufacturer’s instructions. Optical density was measured using photospectrometer Spectramax 340 PC (Molecular Devices) at the wavelength 450 nm.

The represented data show values of two independent analyses normalized to the levels of cytokine expression for cells grown without GAIN scaffolds.

### Ethical issues

Use of all human biological materials for the study was approved by the ethical committee at the National Institute for Health Development, Tallinn, Estonia (permission No. 2234 from December 09, 2010). Written informed consent was obtained from all participants prior to the study. All experiments were performed in accordance with national relevant ethical guidelines and GLP regulations.

## Additional Information

**How to cite this article**: Kazantseva, J. *et al.* Graphene-augmented nanofiber scaffolds demonstrate new features in cells behaviour. *Sci. Rep.* **6**, 30150; doi: 10.1038/srep30150 (2016).
